# Boosting the Power Factor of Benzodithiophene Based Donor–Acceptor Copolymers/SWCNTs Composites through Doping

**DOI:** 10.3390/polym12071447

**Published:** 2020-06-28

**Authors:** Zhongming Chen, Mengfei Lai, Lirong Cai, Wenqiao Zhou, Dexun Xie, Chengjun Pan, Yongfu Qiu

**Affiliations:** 1 School of Environment and Civil Engineering, Dongguan Cleaner Production Technology Center, Dongguan University of Technology, Dongguan 523808, China; clr@dgut.edu.cn; 2 Shenzhen Key Laboratory of Polymer Science and Technology, College of Materials Science and Engineering, Shenzhen University, Shenzhen 518060, China; laimengf026@gmail.com (M.L.); wz37@zips.uakron.edu (W.Z.); pancj@szu.edu.cn (C.P.); 3 School of Chemistry, Sun Yat-sen University, Guangzhou 510275, China; xiedx9@mail.sysu.edu.cn

**Keywords:** organic thermoelectric materials, polymer composites, power factor

## Abstract

In this study, a benzodithiophene (BDT)-based donor (D)–acceptor (A) polymer containing carbazole segment in the side-chain was designed and synthesized and the thermoelectric composites with 50 wt % of single walled carbon nanotubes (SWCNTs) were prepared via ultrasonication method. Strong interfacial interactions existed in both of the composites before and after immersing into the 2,3,5,6-Tetrafluoro-7,7,8,8-tetracyanoquinodimethane (F_4_TCNQ) solution as confirmed by UV-Vis-NIR, Raman, XRD and SEM characterizations. After doping the composites by F_4_TCNQ, the electrical conductivity of the composites increased from 120.32 S cm^−1^ to 1044.92 S cm^−1^ in the room temperature. With increasing the temperature, the electrical conductivities and Seebeck coefficients of the undoped composites both decreased significantly for the composites; the power factor at 475 K was only 6.8 μW m^−1^ K^−2^, which was about nine times smaller than the power factor at room temperature (55.9 μW m^−1^ K^−2^). In the case of doped composites, although the electrical conductivity was deceased from 1044.9 S cm^−1^ to 504.17 S cm^−1^, the Seebeck coefficient increased from 23.76 μV K^−1^ to 35.69 μW m^−1^ K^−2^, therefore, the power factors of the doped composites were almost no change with heating the composite films.

## 1. Introduction

Conjugated polymers (CPs) have been attracting great attention in the field of organic optoelectronics owing to their unique features (e.g., low cost, flexibility, easy processability and low thermal conductivity) [1,2,3,4,5,6]. However, the electrical conductivities (σ) of CPs are generally quite low which hinder their applications to fabricate thermoelectric generators [7,8,9,10]. Their thermoelectric performance is evaluated by the dimensionless figure of merit (ZT), which is defined as ZT = σS^2^ T/ k, where s is the electrical conductivity, S is the Seebeck coefficient, T is the absolute temperature and k is the thermal conductivity [7,8]. Due to the difficulties in measuring the thermal conductivity of organic and/or organic–inorganic composite films, power factor (σS^2^) is used to evaluate the thermoelectric performance instead of ZT for organic thermoelectric materials [10]. However, the power factor cannot solely be used to evaluate the thermoelectric performance. With power factor alone, the thermoelectric performance cannot be fully evaluated. With the development of technology of determining the thermal conductivity of polymer thin films, the thermal conductivity results of the investigated polymers will be released in the future. Polymers generally have larger Seebeck coefficient value(S) compared to inorganic materials, therefore the strategies of improving the electrical conductivities of organic and/or organic–inorganic composites are the key points to achieve organic thermoelectric materials with high performance [11,12,13,14,15].

Doping—which is basically achieved through electron transfer between the CPs backbones and dopants through redox reaction—is one of the most efficient methods to improve the conductivities of CPs [16,17]. We can divide doping into two types, p-doping and n-doping, based on the different doping processes for CPs. In the case of p-doping, the oxidation reaction has happened and positive charge carriers are introduced to the polymer backbones. In contrast, n-doping refers to the addition of electrons to the polymer backbones by reductive reaction [17,18,19,20,21]. As concluded from the previous reports [17,18,19,20,21], it is always welcome to generate charge carriers as many as possible with adding smaller amounts of dopants. Because the addition of large amount of dopants may influence the intense packing of the polymer chains due to the immiscibility of the polymer and dopant, which is generally detrimental to the charge carrier mobility, as a result influencing the electrical conductivities and Seebeck coefficients of the CPs [17,18,19,20,21]. The dopant could be inorganic salt like FeCl_3_ and also could be organic small molecules, such as 2,3,5,6-tetrafluoro-7,7′,8,8′-tetracyanoquinodimethane (F_4_TCNQ), which are found to have superior features to the inorganic salt, such as little alternations of the surface morphology and high doping efficiency, etc. [20]. Chabinyc et al. reported that significantly enhanced power factor value of 120 μWm^−1^ K^−2^ was achieved from the F_4_TCNQ vapor doped conjugated polymer (PBTTT) [21]. Müller et al. reported that after doping the P3HT with F_4_TCNQ vapor, the electrical conductivity could be reached to 12.7 Scm^−1^ [22].

Forming composites with highly conductive inorganic materials (such as single-wall carbon nanotubes (SWCNTs), graphene and graphite, etc.) is another efficient method to combine the large Seebeck coefficients of polymers and high electrical conductivities of inorganic materials simultaneously [23,24,25,26,27,28,29,30]. To date, various kinds of organic/inorganic thermoelectric composites have been developed [23,24,25,26,27,28,29,30], for example, a number of PEDOT/inorganic thermoelectric composites have been reported [24,25,29], Chen et al. fabricated the PEDOT/graphene with a pie shape and the obtained composites showed an excellent power factor of 5.2 ± 0.9 μW m^−1^ K^−2^, which is greater than 13.3 times that of the PEDOT [29]. Yao et al. compositing the poly(3-hexyl)thiophene with SWCNTs and the enhanced power factor (176 μW m^−1^ K^−2^) which was achieved by finely tuning the ordering structure of the polyaniline (PANI) and SWCNTs [30].

The incorporation of the two-dimensional conjugated side-chain to the polymer backbone was confirmed to improve the electrical conductivity of conjugated polymers [31,32,33], thus was widely used as the active layers in the organic-field effect transistors (OFETs) and organic solar cells (OSCs) [34,35]. Recently, we attempted to investigate the thermoelectric performance of BDT-based polymers with two-dimensional conjugated side-chains [36] and obtained good thermoelectric power factor of 101.3 μWm^−1^ K^−2^, which is almost 100 times larger than that of the BDT–EDOT copolymers with one dimensional aliphatic side chain (0.9 μWm^−1^ K^−2^). These results inspired us to further explore the BDT-based copolymers with different conjugated units as the side-chain. Herein, we expand this concept by introducing the carbazole segment into the side-chain to build a two-dimensional BDT-based copolymer bearing conjugated side-chain and composites were prepared after physical mixing of the polymers and SWCNTs and the effect of doping on the thermoelectric properties of polymer composites were systematically investigated. The F_4_TCNQ doped composites exhibited enhanced power factor (64.2 μW m^−1^ K^−1^) than that of non-doped composites and the ability of keeping the power factor in a large temperature range provide new insights on developing high performance TE materials with multiple functions.

## 2. Experimental Section

### 2.1. Raw Materials

The single-walled carbon nanotubes (SWCNTs) (diameter < 3 nm, purity > 95.0 wt %), were purchased from XFNANO Materials Technology Co., Ltd., Nanjing, China, Monomers **M1** and **M2** were purchased from Derthon Optoelectronic Materials Science Technology Co., Ltd., Shenzhen, China, Tetrakis (triphenylphosphine) palladium (Pd(PPh_3_)_4_), P(o-tol)_3_ and MgSO_4_ were purchased from Energy Chemical, Shanghai, China. All commercially available reagents were used directly without further treatment, unless otherwise noted.

### 2.2. Instruments

The ^1^H-NMR spectra of PBDT–C–BT was recorded on an 600 MHz NMR spectrometer (Bruker, Fällanden, Switzerland). Thermal gravimetric analysis (TGA) was conducted on a TGA-55 instrument (TA Instruments, Newcastle, DE, USA) from 25 °C to 700 °C under a 20-mL min^−1^ nitrogen flow and a heating rate of 10 °C min^−1^. The molecular weights and polydispersity index (PDI) of the polymers were determined by size exclusion chromatography (SEC) (Waters e2695 Separations Module, Waters, Singapore) using THF as the eluent and polystyrene as a standard. Ultraviolet-visible-near-infrared (UV-Vis-NIR) absorption spectra were measured using a Lambda 950 spectrophotometer (PerkinElmer, Waltham, MA, USA). The morphology of the polymer films was performed by a SU-70 scanning electron microscope (SEM) (Hitachi SU-70, Tokyo, Japan). The Raman spectra were acquired on a Renishaw in Via-Reflex Raman microscope (Renishaw inVia™ Raman Microscope, London, England). X-ray diffraction (XRD) was measured on a SmartLab X-ray diffractometer (Rigaku, Tokyo, Japan) with a copper target. The electrical conductivity and Seebeck coefficient of the polymer films were recorded by using an MRS-3 thermoelectric test system (Wuhan Joule Yacht Science & Technology, Wuhan, China).

### 2.3. Synthesis of the Polymer

**Scheme 1:**
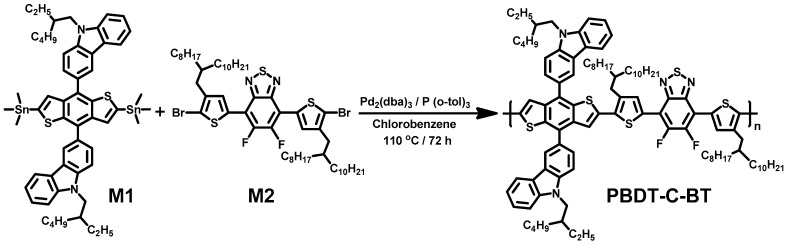
Synthetic procedure of the two-dimensional benzodithiophene (BDT)-based donor (D)–acceptor (A) copolymers (PBDT–C–BT).

As illustrated in Scheme 1, the polymer (PBDT–C–BT) was synthesized using a previously reported method [36], benzodithiophene (**M1**) (210.5 mg, 0.20 mmol) and the benzothiadiazole (**M2**) (207.6 mg, 0.20 mmol), Tris(dibenzylideneacetone)dipalladium (Pd_2_(dba)_3_) (9.0 mg, 0.0098 mmol), Tris(o-toIyl)phosphine (P(o-tol)_3_) (15.0 mg, 0.0492 mmol) were added into a two necked flask, after vacuum and nitrogen for three times, chlorobenzene (8 mL) was injected to the flask via syringe and the reaction mixture was refluxed for 72 h. Then cooled down to room temperature, the mixture was poured into water and extracted with chloroform for three times, the combined organic solutions were dried by magnesium sulfate (MgSO_4_), after filtration and the solvents were removed by distillation, the crude product was then purified by centrifugation (3000 rpm) for 30 minutes to produce PBDT–C–BT as black powder, 291 mg, yield: 93%.

^1^H NMR (600 MHz, chloroform-*d*) *d* 8.54 (d, *J* = 6.5 Hz, 2H), 8.15 (t, *J* = 6.6 Hz, 2H), 8.01 (d, *J* = 10.4 Hz, 2H), 7.89 (t, *J* = 7.3 Hz, 2H), 7.63–7.55 (m, 4H), 7.51–7.42 (m, 6H), 4.43–4.09 (m, 4H), 2.79 (s, 4H), 2.28–2.07 (m, 4H), 1.28–1.14 (m, 80H), 0.95–0.87 (m, 24H). *M*_n_ = 44 kDa, PDI = 1.57.

### 2.4. Preparation of the PBDT–C–BT/SWCNTs Composites

The polymer powders were dissolved in chlorobenzene under vigorously stirring and the stock solution (10 mg/mL) was prepared. In a separate vial, SWCNTs was added into chlorobenzene to form SWCNTS solutions (10 mg/mL) and the SWCNTs were homogeneously dispersing in the chlorobenzene under ultrasonication. The polymer/SWCNTs composites were prepared by mixing the same amounts of the polymer and SWCNTs solutions, after the solutions were completely mixed by ultrasonication, the solutions were casting onto the glass plates to form composites thin films.

### 2.5. Preparation of Doped PBDT–C–BT/SWCNTs Composites

The F_4_TCNQ (11.2 mg) was dissolved in a 2-mL vial with CH_3_CN (0.7 mL) to prepare the dopant solutions. Then the dopant solutions were added to the composites solution to form doped PBDT–C–BT/SWCNTs composites and casted onto the glass plated to form doped PBDT–C–BT/SWCNs composite thin films. We used a 40-KHz sonicator to do sonication of the composites for 24 h. After dipping the sample in the dopant solution, the formed composites films were dried under air at room temperature for 12 h.

## 3. Results and Discussion

### 3.1. Polymer Design, Synthesis and Characterizations

BDT moiety was chosen as the investigation candidate owing to the planarity of the BDT backbone which was confirmed to show good charge carrier mobility [33,34,35]. The thiophene-benzothiadiazole-thiophene was chosen as the comonomer to build the donor–acceptor skeleton of the conjugated backbone. The copolymer was synthesized by using palladium catalyzed Stille-coupling reaction with a good yield of 93% (Scheme 1). The obtained polymer was readily soluble in common organic solvents (e.g., tetrahydrofuran, chloroform, dichloromethane, chlorobenzene, etc.). The number average molecular weight (*M*_n_) and molecular weights distributions (PDI) was determined to be 44 kDa and 1.57, respectively by using size exclusion chromatography (SEC) with THF as the eluent and polystyrene as the standard (Figure S2). The chemical structure of the polymer was unambiguously confirmed by using proton nuclear magnetic resonance (NMR) as shown in Figure S1.

### 3.2. The UV-Vis-NIR Spectra of the Polymer/SWCNTs Composites

**Figure 1:**
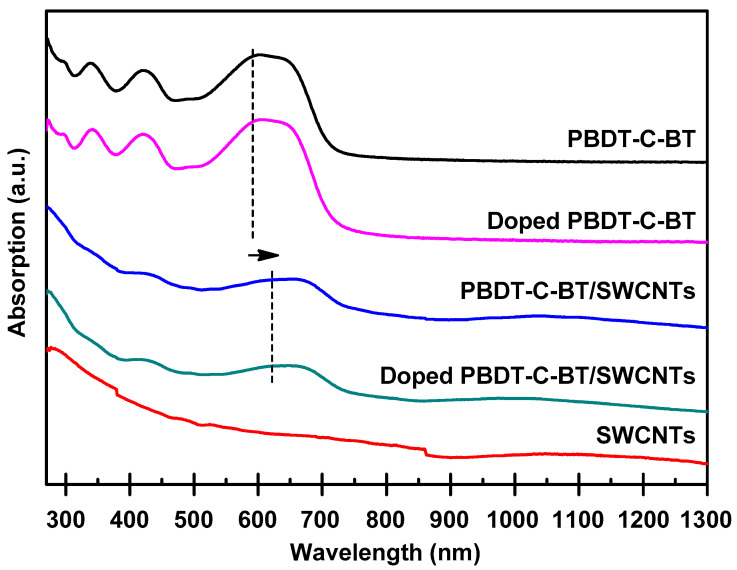
UV-Vis-NIR spectra of the PBDT–C–BT, SWCNTs, PBDT–C–BT/50 wt % SWCNTs (c) and /F_4_TCNQ-doped PBDT–C–BT/50 wt % SWCNTs. All the spectra were recorded in airconditioned room temperature.

In order to explore the electronic structures of the composites, we performed UV-Vis-NIR spectra as shown in Figure 1. The pristine polymer exhibited two obvious peaks at around 421 nm and 600 nm, which should be attributed from the donor–acceptor structure of the polymer backbone, namely, charge transfer was happening from donor unit to the acceptor segment to form the so-called CT band at longer wavelengths region. After composting the polymer with SWCNTs with a :1 ratio, the intensities of the peaks at around 450 nm and 600 nm decreased due to the successfully combination of the spectra of SWCNTs and the peak at 603 nm for pristine polymer film slightly shifted to longer wavelength (653 nm) after forming composite films, confirming the interfacial interactions between the SWCNTs and polymers existed. Only small changes of the spectra were observed after doping the pristine polymer by F_4_TCNQ, suggesting the inefficient doping of the pristine polymers. After forming polymer/SWCNTs composites, a new peak appeared at 1100 nm due to the indicating the successfully formation of polaron. Though the reason was still unclear at current stage, this phenomena further indicated the existed strong interactions between the polymer and SWCNTs. The doped polymer/SWCNTs composites also showed obvious shift of the spectra due to the interfacial interactions between the polymer and SWCNTs. These results indicated that the polymer and SWCNTs could be tightly mixed with each other through π-π stacking.

### 3.3. Raman Spectra of the Polymer/SWCNTs Composites

**Figure 2:**
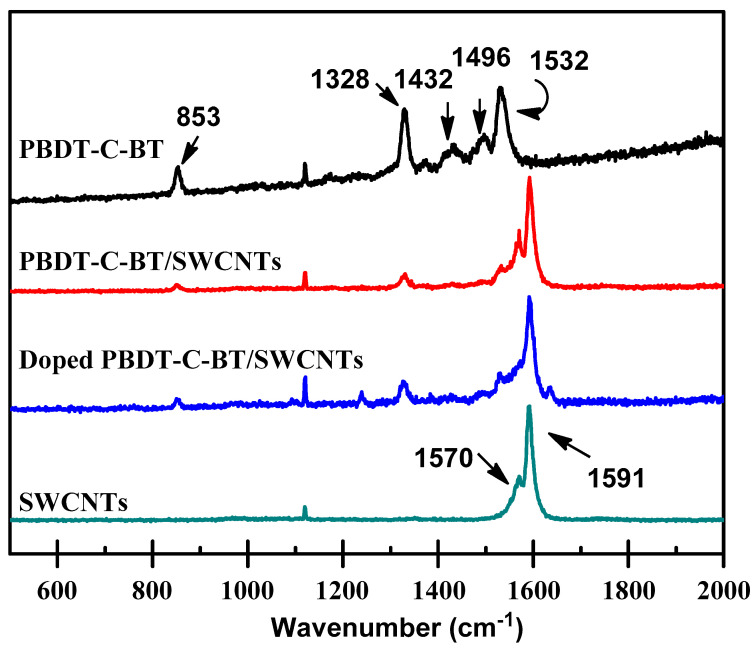
Raman spectra of PBDT–C–BT, SWCNTs, PBDT–C–BT/50 wt % SWCNTs and /F_4_TCNQ doped PBDT–C–BT/50 wt % SWCNTs.

Raman spectra is a commonly used strategy to investigate the interfacial interactions of composites. As shown in Figure 2, Raman spectra were acquired for the polymer, PBDT–C–BT/SWCNTs composites and PBDT–C–BT/SWCNTs composites doped with F_4_TCNQ and SWCNTs. The peak at 853 cm^−1^ should be ascribed to the stretching vibration of C–F bond on the benzothiadiazole unit and the stretching vibration peak of C–C bond should be 1328 cm^−1^ and the peak of 1432 cm^−1^ belongs to the symmetrically vibration of the C=C bond on the thiophene ring, the peaks at 1496 cm^−1^ and 1532 cm^−1^ should be contributed from the vibration of the BT and BDT segments, respectively. After forming the composites, 2–4-nm shifts of the peaks were observed from pristine polymer to the PBDT–C–BT/SWCNTs composites, indicating the strong interactions existed between the polymer and SWCNTs, similar phenomena are also found in the doped PBDT–C–BT/SWCNTs composites, furthermore, new peaks at 1243 cm^−1^, 1388 cm^−1^ and 1640 cm^−1^ appeared after doping the composites, confirming the successful formation of quinoid structure in PBDT–C–BT backbone (Figure S4).

### 3.4. Surface Morphology of the Composites

**Figure 3:**
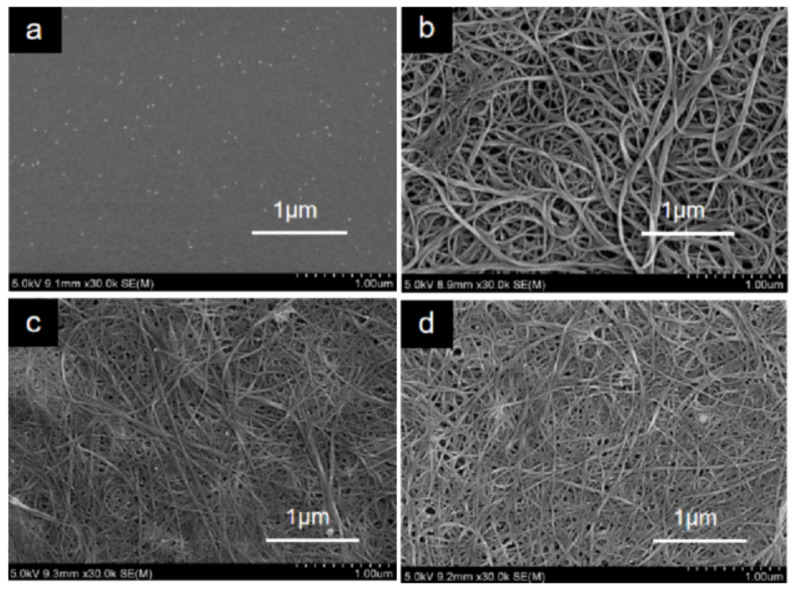
SEM images of (**a**) PBDT–C–BT film, (**b**) SWCNTs, (**c**) PBDT–C–BT/50 wt % SWCNTs and (**d**) /F_4_TCNQ-doped PBDT–C–BT/50 wt % SWCNTs.

The surface morphologies of the composites can influence the charge carrier transport, thus affecting the electrical conductivities and Seebeck coefficient, as a result, also affecting the TE performance. As shown in Figure 3, the SEM image (Figure 3a) of the pristine polymer exhibited a flat plane morphology, only very small particles were observed. The pure SWCNTs showed a fibrillar images as shown in Figure 3b. After forming the composites with SWCNTs, homogeneous fibrillar images were also obtained for PBDT–C–BT/SWCNTs composites before and after doping with F_4_TCNQ. We hypothesize that the SWCNTs and polymer chains were bounded tightly with each other. In order to confirm this hypothesis, we conducted TGA experiments as shown in Figure S3, the 5% weight loss temperatures for the polymer (375 °C), polymer composites (375 °C), doped polymer composites (360 °C) were almost identical, such a similar degradation procedure indicated that the formation of composites had no effect on the thermal stabilities of the materials. The XRD spectra were conducted to investigate the microstructures of the PBDT–C–BT/SWCNTs composites before and after doping is presented in Figure S5. A halo peak was observed ranging from 15° to 40°, indicating that the introduced two-dimensional conjugated side-chain deteriorated the conjugated backbone to form an intensified packing structure, thus the obtained polymer showed the amorphous feature in the thin-film state.

### 3.5. Thermoelectric Properties of the Composites

**Figure 4:**
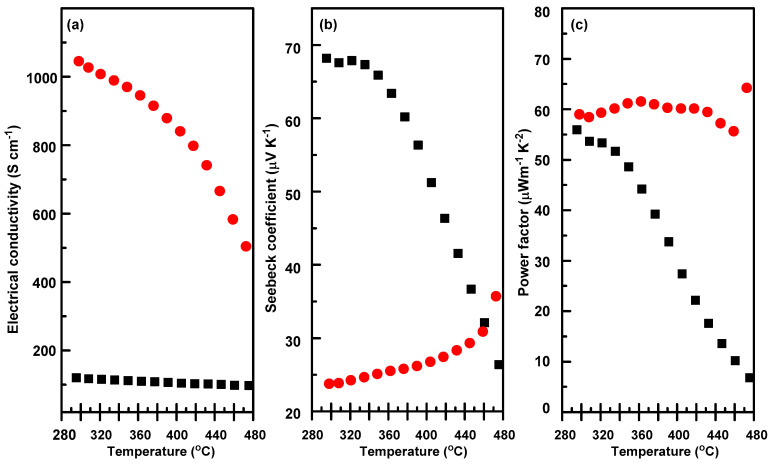
(**a**) Electrical conductivity, (**b**) Seebeck coefficient and (**c**) power factor of the pristine composites (black) and F_4_TCNQ-doped composites (red).

We performed the temperature dependent thermoelectric properties study of the PBDT–C–BT/SWCNTs (50 wt %) composites before and after doping with F_4_TCNQ. We also attempted to test the thermoelectric performances of PBDT–C–BT only before and after doping with F_4_TCNQ, but the values are too small to record. As shown in Figure 4a, the electrical conductivity of composite is keeping constant (around 120 Scm^−1^) as increasing the temperature. Not like the trend of the electrical conductivity, the Seebeck coefficients of the composites are becoming smaller with increasing the temperature, suggesting that there may be different types of charge carriers (polarons or bipolarons) in different temperatures. However, the doped PBDT–C–BT/50 wt % SWCNTs composite exhibited a much enhanced electrical conductivity of 1005 S cm^−1^ at room temperature, which is almost 5 times in magnitude higher than that of PBDT–C–BT/50 wt % SWCNTs. Interestingly, the electrical conductivity of the doped composites was gradually decreasing as enhancing the temperature, which may be due to the de-doping process of F_4_TCNQ. The smaller Seebeck coefficient values of the doped composites were noticed (Figure 4b), which means the doping of the composites mainly increase the carrier concentration, which is detrimental to the values of Seebeck coefficients. In the end, the power factor of the doped composites almost keep as a constant (around 60 μWm^−1^ K^−2^) in the whole temperature range, however, the power factor value of the PBDT–C–BT/SWCNTs composites significantly dropped from around 56 mWm^−1^ K^−1^ at room temperature to less than 7 μWm^−1^ K^−2^ at 480 K. The electrical conductivity, Seebeck coefficient and power factor of pristine SWCNTs were recorded and shown in Figure S6. As we can see, the electrical conductivity of SWCNTs could reach to 1146.5 Scm^−1^, therefore the charge carrier should mainly transport through the SWCNTs. These results indicated that chemically doping the PBDT–C–BT/SWCNTs is an efficient method to enhance the thermoelectric performance, the interestingly keeping of the power factor in such a large temperature range may also have the potential to fabricate the organic thermoelectric generator which could be used in a large temperature range.

## 4. Conclusions

A new type of thermoelectric composites was prepared by forming a two-dimensional BDT-based D–A copolymer and SWCNTs composites and F_4_TCNQ was used as a chemical dopant to dope the composites. The properties of the polymer composites before and after doping with F_4_TCNQ were systematically investigated by UV-Vis-NIR, SEM, Raman and XRD characterizations. It was found that strong interfacial interactions existed in the polymer composites before and after doping. Interestingly, the electrical conductivity of the doped polymer composites showed almost 20 times higher than that of the non-doped the composites in the room temperature and the power factors of the doped polymer composites is almost no change from 290 K to 480 K, which is always larger than those of the non-doped composites in the whole temperature range. These results indicate that chemically doping of the conjugated/SWCNTs composites is a promising strategy to achieve high thermoelectric performance.

## Supplementary Materials

The following are available online at https://www.mdpi.com/2073-4360/12/7/1447/s1, Figure S1:^1^H NMR spectrum of PBDT–C–BT, Figure S2: GPC curve of PBDT–C–BT, Figure S3: TGA curves of PBDT–C–BT, Figure S4: Expanded RAMAN spectra of pristine polymer composite (black) and doped polymer composites (red), Figure S5: XRD spectra of polymer, SWCNTs, pristine polymer composite and doped polymer composites, Figure S6: Electrical conductivity, Seebeck coefficient, and power factor of the pristine SWCNTs.
