# Supplementary material for: Boosting the Power Factor of Benzodithiophene Based Donor–Acceptor Copolymers/SWCNTs Composites through Doping
Source: Polymers (Basel). 2020 Jun 28;12(7):1447. doi: 10.3390/polym12071447 (PMC7407128; doi:10.3390/polym12071447)
Supplement: Supplementary file 1 [file polymers-12-01447-s001.pdf]

Supporting information

for

Boosting the Power Factor of Benzodithiophene based Donor–Acceptor Copolymers/SWCNTs Composites through Doping

Zhongming Chen [1,*], Mengfei Lai [2], Lirong Cai [1], Wenqiao Zhou [2], Dexun Xie [3] , Chengjun Pan [2] and Yongfu Qiu [1,*]

a) School of Environment and Civil Engineering, Dongguan Cleaner Production Technology Center, Dongguan University of Technology, Guangdong 523808, P. R. China.

b) Shenzhen Key Laboratory of Polymer Science and Technology, College of Materials Science and Engineering, Shenzhen University, Shenzhen 518060, China.

c) School of Chemistry, Sun Yat-sen University, Guangzhou, 510275, China.

Correspondence: zmchen@dgut.edu.cn (Z.C.); qiuyf@dgut.edu.cn (Y.Q.)

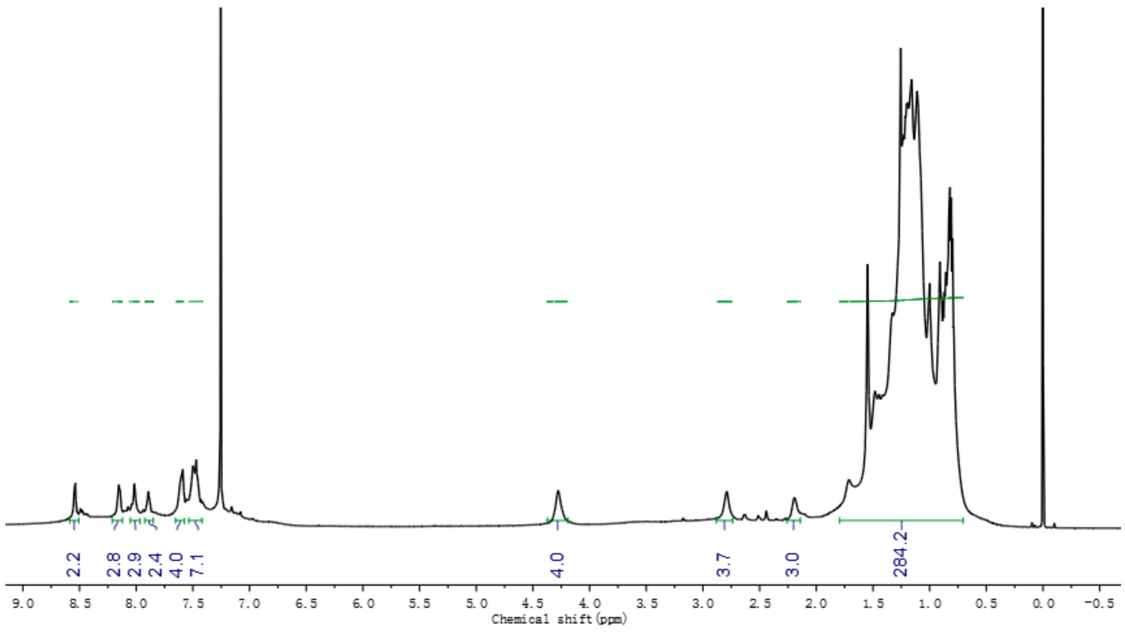

Figure S1. ^{1}H NMR spectrum of the polymer.

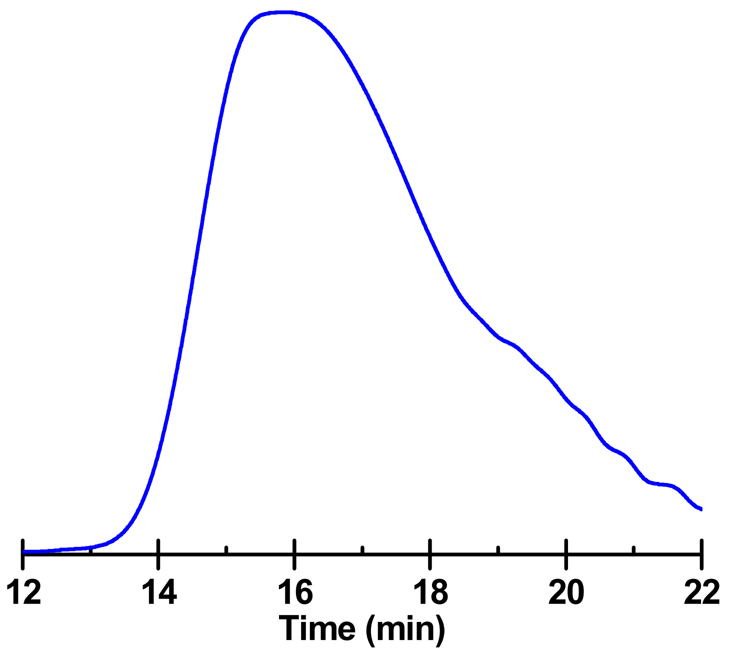

Figure S2. GPC curve of the polymer

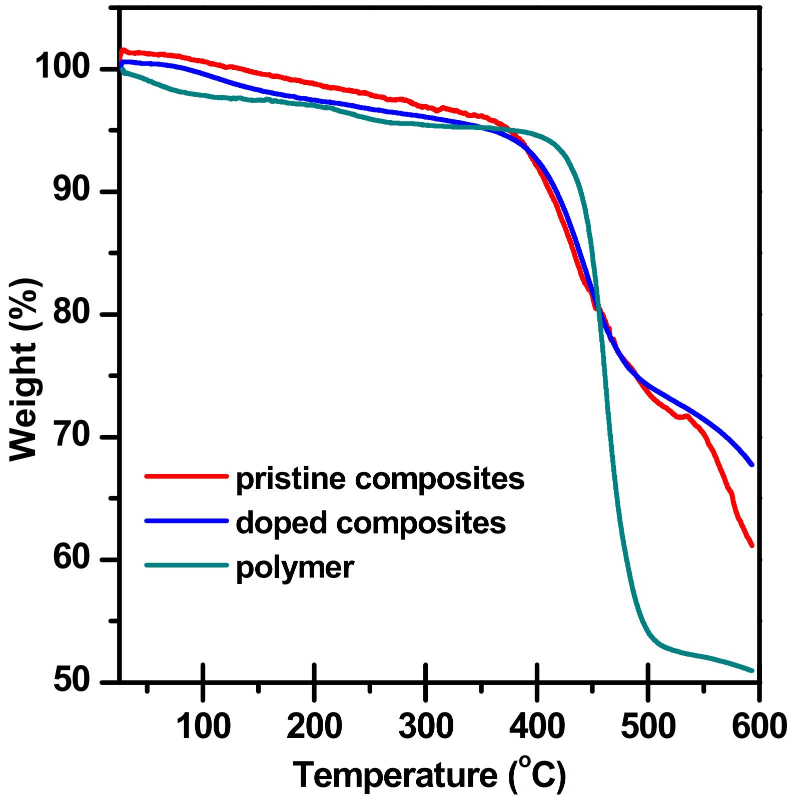

Figure S3. TGA curves of the polymer, pristine composites, and doped composites.

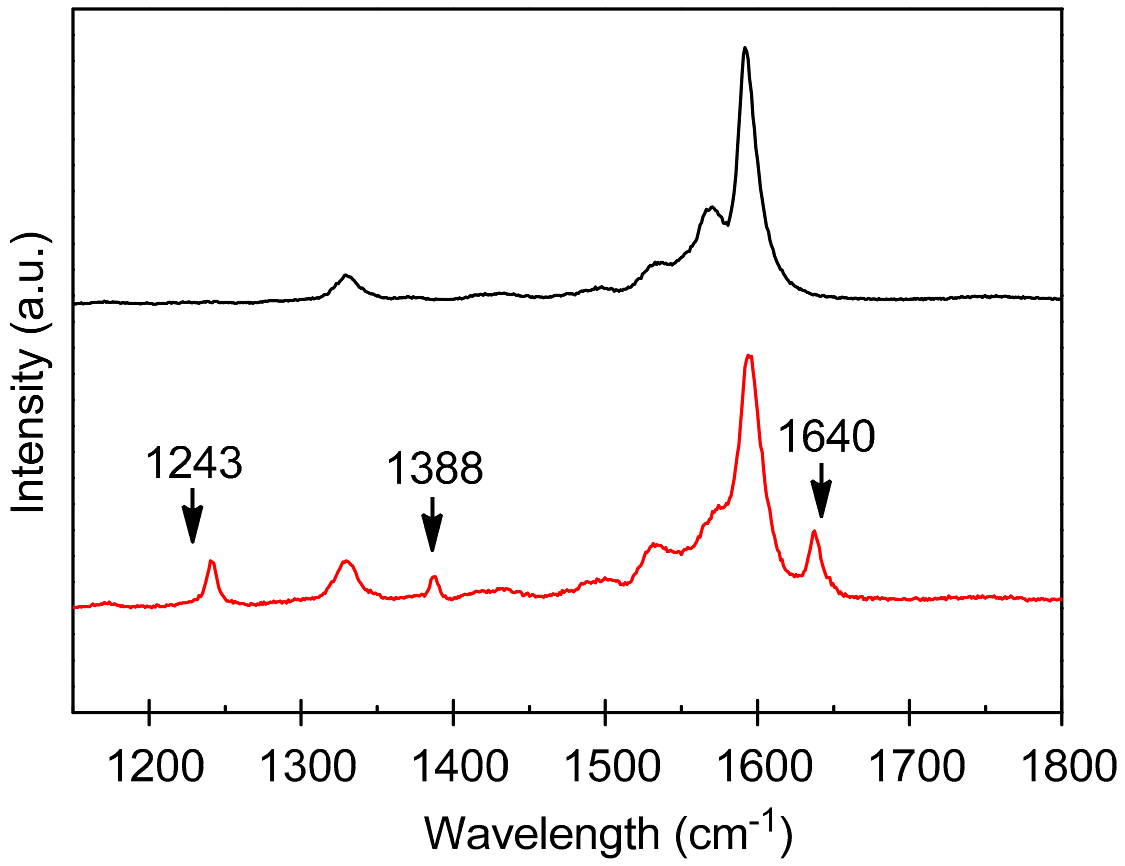

Figure S4. Expanded RAMAN spectra of pristine polymer composite (black) and doped polymer composites (red).

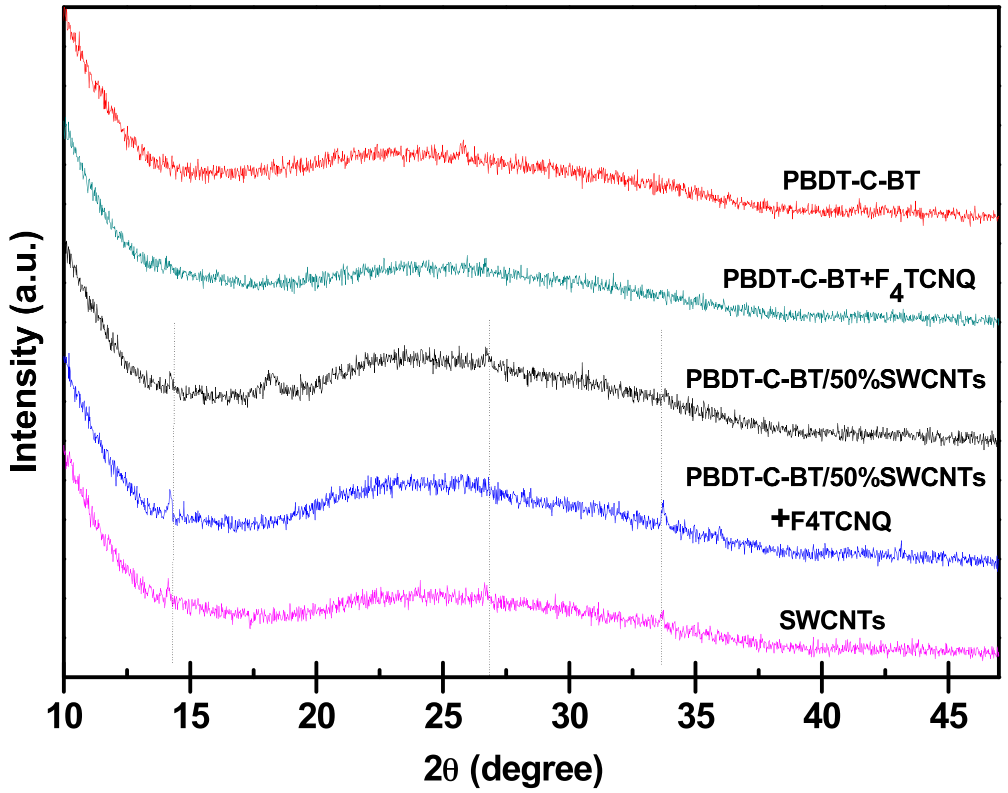

Figure S5. XRD spectra of polymer, SWCNTs, pristine polymer composite, and doped polymer composites .

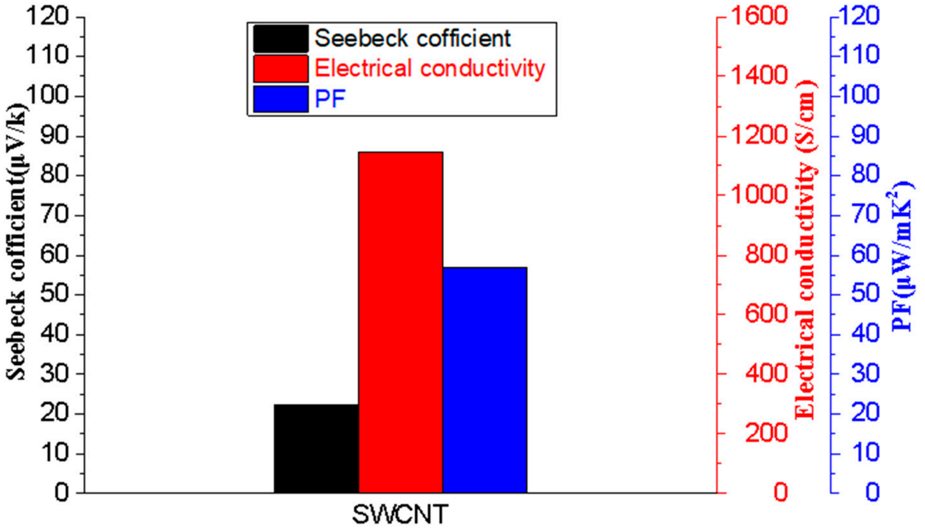

Figure S6. Electrical conductivity, Seebeck coefficient, and power factor of the pristine SWCNTs.
